# Supplementary material for: Predicting Anticancer Drug Resistance Mediated by Mutations
Source: Pharmaceuticals (Basel). 2022 Jan 24;15(2):136. doi: 10.3390/ph15020136 (PMC8878306; doi:10.3390/ph15020136)
Supplement: Supplementary file 1 [file pharmaceuticals-15-00136-s001.zip › pharmaceuticals-1557619-supplementary/TableS1.pdf]

SUPPLEMENTARY INFORMATION

Table S1. The list of 45 feature vectors used for training.

#	**Category**	**Abbreviation**	**Feature name**
1	Interaction energy	DKE-ele	Docking energy: electrostatic
2	Interaction energy	DKE-hb	Docking energy: hydrogen-bonding
3	Interaction energy	DKE-vdw	Docking energy: van der Waals forces
4	Microenvironment	WCN-cα	WCN: C_α atoms in SAV chain
5	Microenvironment	WCN-n	WCN: Nitrogen atoms in SAV chain
6	Microenvironment	WCN-o	WCN: Oxygen atoms in SAV chain
7	Microenvironment	W-H-pol	WCN: *H*-polar (RKEDQN)
8	Microenvironment	W-H-neu	WCN: *H*-neutral (GASTPHY)
9	Microenvironment	W-H-hyd	WCN: *H*-hydrophobic (CVLIMFW)
10	Microenvironment	W-V-sma	WCN: *V*-small (GASCTPD)
11	Microenvironment	W-V-med	WCN: *V*-medium (NVEQIL)
12	Microenvironment	W-V-lar	WCN: *V*-large (MHKFRYW)
13	Microenvironment	W-Z-low	WCN: *Z*-low polarizability(GASDT)
14	Microenvironment	W-Z-med	WCN: *Z*- medium polarizability (CPNVEQIL)
15	Microenvironment	W-Z-hig	WCN: *Z*-high polarizability (KMHFRYW)
16	Microenvironment	W-P-low	WCN: *P*-low polarity (LIFWCMVY)
17	Microenvironment	W-P-neu	WCN: *P*-neutral polarity (PATGS)
18	Microenvironment	W-P-hig	WCN: *P*-high polarity (HQRKNED)
19	Microenvironment	W-F-cha	WCN: *F*-charged (DEHKR)
20	Microenvironment	W-F-pol	WCN: *F*-polar (CGNQSTY)
21	Microenvironment	W-F-npo	WCN: *F*-nonpolar (AFILMPVW)
22	Microenvironment	W-E-aci	WCN: *E*-acidic (DE)
23	Microenvironment	W-E-bas	WCN: *E*-basic (HKR)
24	Microenvironment	W-E-aro	WCN: *E*-aromatic (FWY)
25	Microenvironment	W-E-ami	WCN: *E*-amide (NQ)
26	Microenvironment	W-E-shy	WCN: *E*-small hydroxyl (ST)
27	Microenvironment	W-E-sul	WCN: *E*-sulfur-containing (CM)
28	Microenvironment	W-E-ap1	WCN: *E*-aliphatic 1 (AGP)
29	Microenvironment	W-E-ap2	WCN: *E*-aliphatic 2 (ILV)
30	Structure characteristics	BFT	B-factor of SAV

31	Structure characteristics	RSA	Related solvent accessibility
32	Structure characteristics	SSE	Secondary structure elements
33	Structure characteristics	EHB-acc	Energy of backbone H-bond: acceptor
34	Structure characteristics	EHB-don	Energy of Backbone H-bond: donor
35	Sequence conservation	SSI-b62	Substitution index: BLOSUM62
36	Sequence conservation	SSI-p250	Substitution index: PAM250
37	Sequence conservation	SSI-pssm	Substitution index: PSSM
38	Sequence conservation	ETP-sav	Entropy: SAV
39	Sequence conservation	ETP-avg3	Entropy: Average of 3 residues
40	Sequence conservation	ETP-avg5	Entropy: Average of 5 residues
41	Sequence conservation	ETP-avg7	Entropy: Average of 7 residues
42	Sequence conservation	ETP-avg9	Entropy: Average of 9 residues
43	Sequence conservation	ETP-avg11	Entropy: Average of 11 residues
44	Sequence conservation	ETP-avg13	Entropy: Average of 13 residues
45	Sequence conservation	ETP-avg15	Entropy: Average of 15 residues
